# Clonality of HTLV-2 in Natural Infection

**DOI:** 10.1371/journal.ppat.1004006

**Published:** 2014-03-13

**Authors:** Anat Melamed, Aviva D. Witkover, Daniel J. Laydon, Rachael Brown, Kristin Ladell, Kelly Miners, Aileen G. Rowan, Niall Gormley, David A. Price, Graham P. Taylor, Edward L. Murphy, Charles R. M. Bangham

**Affiliations:** 1 Section of Immunology, Imperial College London, Wright-Fleming Institute, London, United Kingdom; 2 Institute of Infection and Immunity, Cardiff University School of Medicine, Cardiff, United Kingdom; 3 Illumina, Little Chesterford, Essex, United Kingdom; 4 Section of Infectious Diseases, Imperial College London, Wright-Fleming Institute, London, United Kingdom; 5 Departments of Laboratory Medicine and Epidemiology/Biostatistics, University of California San Francisco and Blood Systems Research Institute, San Francisco, California, United States of America; University of Pennsylvania School of Medicine, United States of America

## Abstract

Human T-lymphotropic virus type 1 (HTLV-1) and type 2 (HTLV-2) both cause lifelong persistent infections, but differ in their clinical outcomes. HTLV-1 infection causes a chronic or acute T-lymphocytic malignancy in up to 5% of infected individuals whereas HTLV-2 has not been unequivocally linked to a T-cell malignancy. Virus-driven clonal proliferation of infected cells both in vitro and in vivo has been demonstrated in HTLV-1 infection. However, T-cell clonality in HTLV-2 infection has not been rigorously characterized. In this study we used a high-throughput approach in conjunction with flow cytometric sorting to identify and quantify HTLV-2-infected T-cell clones in 28 individuals with natural infection. We show that while genome-wide integration site preferences in vivo were similar to those found in HTLV-1 infection, expansion of HTLV-2-infected clones did not demonstrate the same significant association with the genomic environment of the integrated provirus. The proviral load in HTLV-2 is almost confined to CD8^+^ T-cells and is composed of a small number of often highly expanded clones. The HTLV-2 load correlated significantly with the degree of dispersion of the clone frequency distribution, which was highly stable over ∼8 years. These results suggest that there are significant differences in the selection forces that control the clonal expansion of virus-infected cells in HTLV-1 and HTLV-2 infection. In addition, our data demonstrate that strong virus-driven proliferation *per se* does not predispose to malignant transformation in oncoretroviral infections.

## Author Summary

The two human retroviruses HTLV-1 and HTLV-2 are similar in their structure, replication cycle and the manner through which they spread between and within individuals. They differ in their preferred host T-cell type and in their possible clinical outcomes. HTLV-2 has not been linked with a specific disease, whereas HTLV-1 infection can cause leukemia and profound neuropathology. It is well established that HTLV-1-infected cells undergo clonal expansion in infected individuals, but little is known about clonality in HTLV-2 infection. In this work, we demonstrate that the extent of HTLV-2-infected cell expansion significantly exceeds that of HTLV-1-infected cells in healthy carriers, approximating instead to that observed in patients with HTLV-1-associated leukemia. Furthermore, we show that HTLV-2 characteristically resides in a small number of expanded clones that persist over time, and that the degree of oligoclonality significantly correlates with viral burden in HTLV-2-infected individuals. These results highlight the distinction between in vivo clonal proliferation and malignant transformation, and suggest that the infected cell type may be a more important determinant of clinical outcome in retroviral infections.

## Introduction

The retroviruses HTLV-1 and HTLV-2 diverged from each other more than one million years ago [1] before becoming established in humans. They are similar in several crucial respects, with homologous genome structures that encode a number of regulatory proteins, including the pro-proliferative gene *tax* [2], [3]. Both viruses are transmitted by transfer of infected lymphocytes via breast feeding, blood transfusion and sexual contact [4]. However, their geographical distributions are quite different. HTLV-1 is endemic in particular regions of Japan, sub-Saharan Africa, the Caribbean and South America [5], whereas HTLV-2 is primarily endemic in indigenous populations in Africa and the Americas, although it can also be found among injection drug users in Europe and the United States [4].

HTLV-1 causes both inflammatory and lymphoproliferative diseases. In contrast, HTLV-2 causes little disease. By following a large cohort of HTLV-1/2-infected and seronegative individuals for almost two decades, the HTLV Outcomes study (HOST) detected myelopathy and other neurologic abnormalities among HTLV-2-infected subjects [6], [7], a finding supported by other studies [8]. HTLV-2 was also associated with an increase in both all-cause and cancer-related mortality [9], as well as persistently elevated lymphocyte and platelet counts, suggesting chronic low-level inflammation [10]. However, no mechanistic inferences can yet be drawn.

An important distinction between HTLV-1 and HTLV-2 lies in their host cell predilection. Although they use the same cellular receptors [11], HTLV-1 preferentially infects CD4^+^ T-cells, whereas HTLV-2 favours CD8^+^ T-cells [12], [13]. The biological basis for this difference is not clear. In vitro evidence suggests that the relative cell surface density of two host receptors, heparan sulphate proteoglycans and glucose-transporter 1 [14], determines host cell tropism. However, in vivo studies suggest that both T-cell lineages are efficiently infected by both viruses, and that subsequent proliferation of CD4^+^ or CD8^+^ T-cells driven by HTLV-1 or HTLV-2, respectively, leads to differential expansion of the two T-cell subsets [15].

It is known that HTLV-2, like HTLV-1, can immortalize human lymphocytes in culture [16], [17]. Both HTLV-1 [18], [19] and HTLV-2 [20], [21] have also been shown to cause selective proliferation of certain infected T-cell clones in vivo. Although the molecular pathways by which the viral proteins drive cellular proliferation are well described [22], the mechanistic basis of selective clonal proliferation is not understood. We have recently shown that the genomic integration site and transcriptional orientation of the provirus relative to the nearest host gene play important roles in determining selective HTLV-1 clonal abundance in vivo [23], [24]. However, the total number of infected clones in a single host has not been accurately determined until recently. It was previously estimated that the number of clones in a typical HTLV-1-infected host was of the order of 100 [25], and that individuals with the inflammatory disease HAM/TSP had a smaller number of more abundant clones, i.e. a more oligoclonal distribution. However we have shown [23], [24] (Laydon et al., manuscript submitted) that the total number of clones is in fact 100-fold to 1,000-fold greater (median 28,000), and that patients with HAM/TSP differ from asymptomatic carriers in that they have a larger number of clones rather than a more oligoclonal distribution. In HTLV-2 infection, neither the number nor the absolute or relative abundance of infected T-cell clones has been quantified. It has been suggested that the greater in vitro IL-2 dependency of HTLV-2-infected cells might lead to decreased clonal proliferation in vivo, which might in turn explain the difference in oncogenic potential between the two viruses [26].

In this work, we investigated natural HTLV-2 infection by quantifying the viral burden in CD4^+^ and CD8^+^ T-cells, comparing the clonal distribution of HTLV-2-infected peripheral blood mononuclear cells (PBMCs) to that observed in HTLV-1, and examining the genomic environment of integrated HTLV-2 proviruses. For these purposes, we adapted our recently described high-throughput method for the identification, mapping and quantification of retroviral integration sites, which we have used previously to study host factors associated with clonal abundance, proviral expression and disease progression by tracking infected clones using the genomic coordinates of retroviral integration sites [24], [27]–[29] (Hodson et al., manuscript submitted). This method also allows us to calculate, using the Gini index [30], the degree of oligoclonality and the relative in vivo clonal expansion of infected clones.

## Results

### HTLV-2 is largely restricted to CD8^+^ T-cells

To determine the relative contributions of CD4^+^ and CD8^+^ T-cells to the HTLV-2 proviral load, PBMCs from 28 HTLV-2-seropositive carriers were sorted by flow cytometry into separate CD3^+^ CD4^+^CD8^−^ and CD3^+^CD4^−^CD8^+^ populations. Integration sites were then mapped and quantified in DNA extracted from both sorted and unsorted cells using our previously described [23] method.

We assumed that HTLV-2 infects a single-positive CD4^+^CD8^−^ or CD4^−^CD8^+^ T-cell, and attributed the integration sites found in the unsorted sample to either CD4^+^ or CD8^+^ cells based on the sorted sample in which they were found more frequently. Across all samples, 50% of sites (representing >99% of proviruses) could be attributed in this manner.

**Figure 1:**
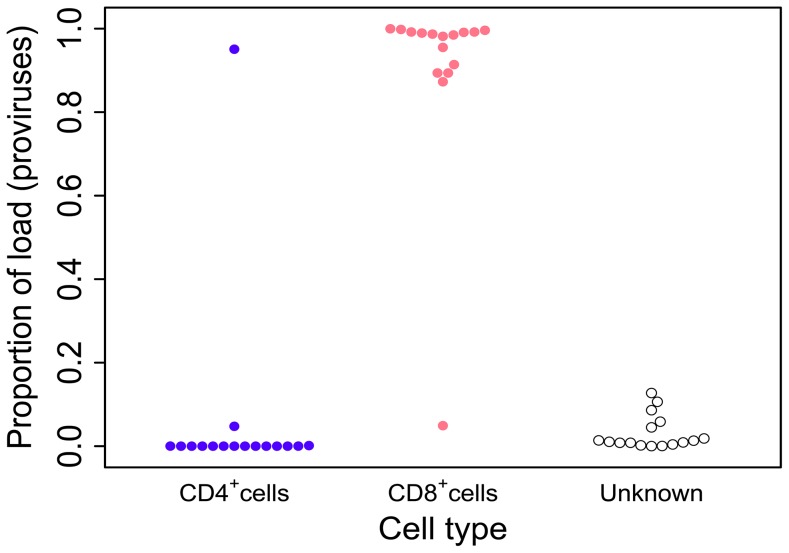
HTLV-2 infection is found almost exclusively in CD8^+^ T-cells. Cryopreserved PBMCs from 28 HTLV-2-infected individuals were sorted by flow-cytometry into separate CD3^+^CD4^+^CD8^−^ and CD3^+^CD4^−^CD8^+^ cell populations. Integration site content was determined by high-throughput sequencing for both sorted populations and unsorted PBMCs. In the unsorted cells, integration sites were positively assigned to CD4^+^ or CD8^+^ cells based on the sorted fraction in which the same sites were found. The proportion of the load was calculated as the sum of the relative frequencies of the clones. Unknown – proportion of the load made up by clones that were not resampled in either the CD4^+^ or CD8^+^ fraction. Since the redetection of clones is most unlikely if proviral load is very low, only individuals in whom >100 proviruses were found are shown here.

Although the number of CD4^+^ cells isolated by flow sorting exceeded the number of CD8^+^ cells in the majority of samples, HTLV-2 was identified chiefly in the CD8^+^ fraction, whether quantified as the number of sequencing reads or the number of proviruses (Supplementary Figure S1). In 15 out of 16 patients (those with sufficiently high numbers of detected proviruses) the HTLV-2 load was almost wholly confined to CD8^+^ cells (mean = 96.3%,median = 98.7%); a mean of only 0.3% (median = 0%) was positively attributed to CD4^+^ cells (Figure 1). In the remaining individual, the majority of the HTLV-2 load was found in CD4^+^ cells.

### The genomic environment of integrated HTLV-2 provirus

**Figure 2:**
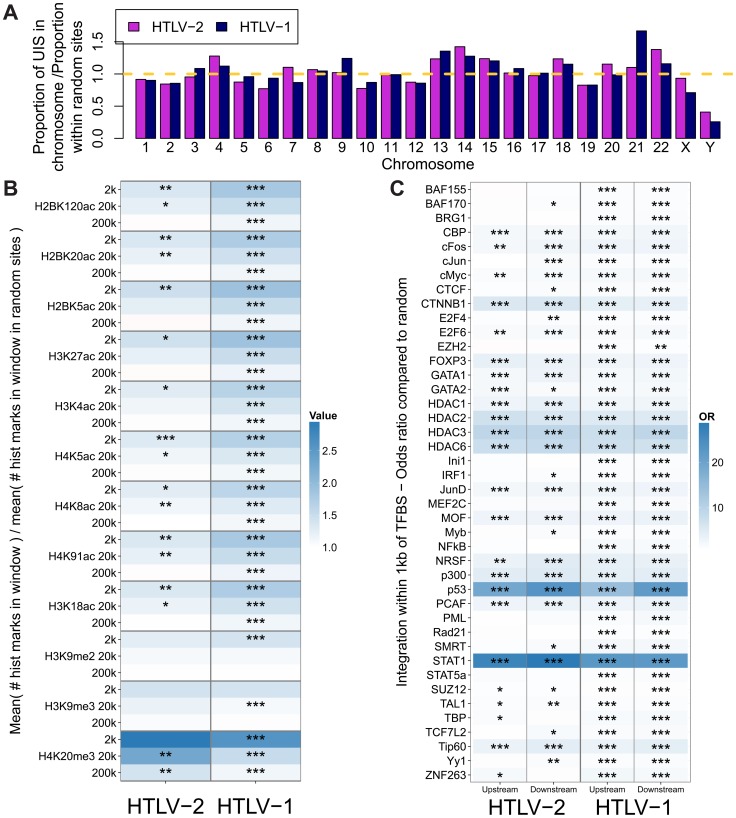
HTLV-1 and HTLV-2 integrate in similar genomic environments. Cells from 28 HTLV-2-infected and 16 HTLV-1-infected subjects were tested for genomic integration site preferences. (A) The ratio of the proportion of sites found in each chromosome (out of the total integration sites found for each virus) to the proportion of randomly generated (in silico) sites in the same chromosome. The yellow dashed line represents random sites (ratio = 1). (B) The number of histone marks (post-translational modifications) in three given windows across integration sites (for example, the 2k window incorporates 1,000 bases on either side of an integration site) compared to the number of histone marks in the same window across random sites. Statistical significance was assessed using the two-tailed Mann-Whitney test (* <0.05, ** <0.01, *** <0.001). (C) The odds ratio of integration within 1 kb of given ChIP-seq sites compared to random sites. The terms upstream and downstream here refer to the 5′ and 3′ sides of the integrated provirus, respectively. Statistical significance was assessed using Fisher's exact test (* <0.05, ** <0.01, *** <0.001).

We analysed proviral integration sites in PBMCs isolated from 28 HTLV-2-infected individuals and 16 HTLV-1-infected individuals without malignant disease. At the nucleotide level, the consensus sequence flanking HTLV-2 genomic integration sites was very similar to that reported for HTLV-1 infection [31], with bias towards GT at positions -3 and -2, respectively, and AC at positions 8 and 9, respectively, across the 6 base repeat (Supplementary Figure S2). The chromosomal distribution of integration sites was similar for HTLV-1 and HTLV-2 (Figure 2A): In each case the frequency of integration sites detected in certain chromosomes in vivo was remarkably greater (e.g. chromosome 13) or lower (e.g. chromosome 10) than expected by chance.

HTLV-1 and HTLV-2 were also similar with respect to features of the genomic environment flanking the provirus. In particular, activating and repressive histone marks were similarly enriched at integration sites compared to random expectation for both viruses (Figure 2B). Previously, we found that HTLV-1 integration was significantly more frequent than expected on a random basis in proximity to ChIP-seq-verified binding sites for certain transcription factors and chromatin modifying proteins, most notably STAT-1, p53 and HDACs [24]. In vivo, proviruses also lie near these binding sites more frequently than expected by chance. In the present study, we reproduced this observation in an independent cohort of HTLV-1-infected individuals, and observed a similar integration targeting preference for HTLV-2 (Figure 2C). Although the magnitude of bias toward these genomic sites was very similar for both viruses, statistical significance was lower in the case of HTLV-2, most likely owing to the lower number of total integration sites.

### HTLV-2 integration is characterized by small numbers of expanded clones

**Figure 3:**
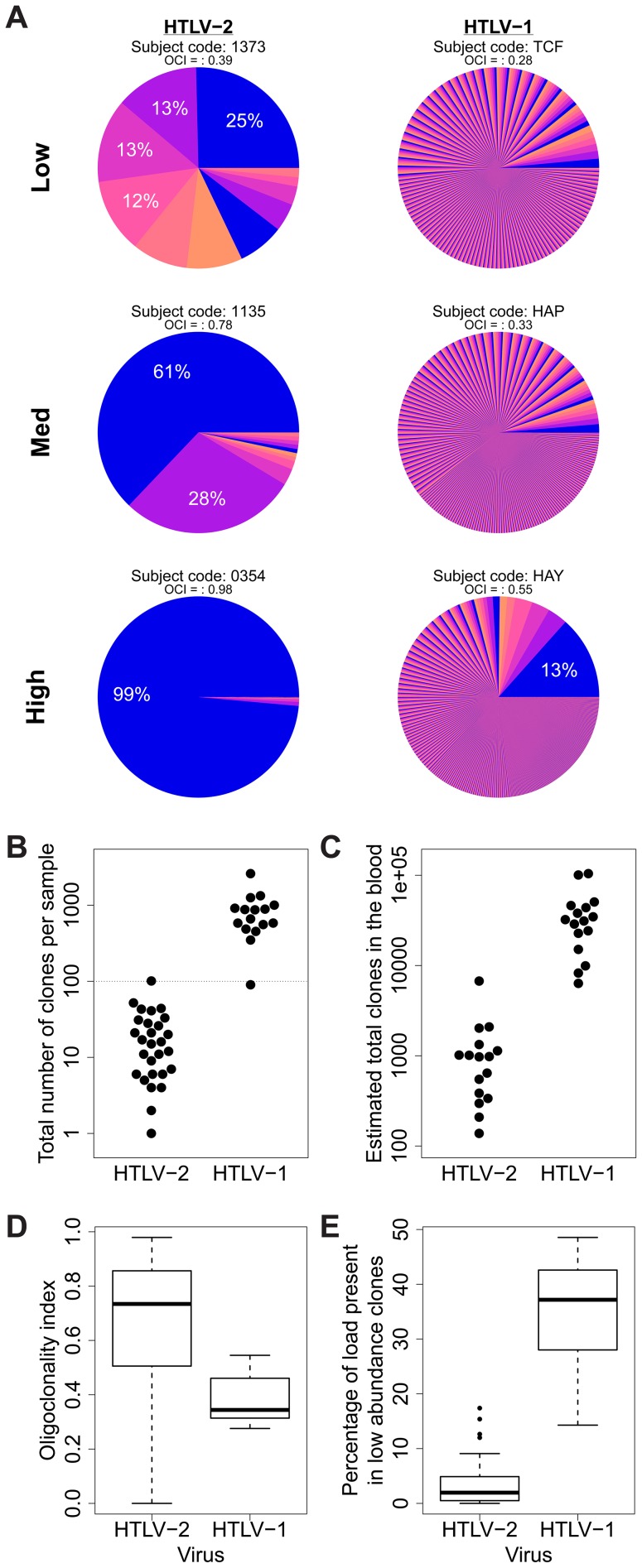
HTLV-2 integration is highly oligoclonal, characterized by small numbers of expanded clones. (A) Clonal distribution in representative subjects with HTLV-1 or HTLV-2 infection. The lowest observed, median and highest observed oligoclonality index values are shown. Each pie slice represents a single clone, proportional to relative abundance. Subjects with >100 proviruses identified are shown. OCI = oligoclonality index. (B) The observed number of clones in each subject with HTLV-1 or HTLV-2 infection (p<0.001, Mann-Whitney test). (C) The total number of clones in the blood was estimated using the *DivE* estimator (Laydon et al., manuscript submitted). Only samples containing sufficient information are shown. For each subject, the population size of infected cells in the blood was estimated based on the proviral load and average PBMC count. The estimated total number of clones in the blood was between 1 and 2 orders of magnitude lower in HTLV-2-infected subjects than in HTLV-1-infected subjects (p<0.001, Mann-Whitney test). (D) The oligoclonality index across all HTLV-1 -infected subjects compared to HTLV-2-infected subjects (p<0.001, Mann-Whitney test). (E) The percentage of the load maintained by clones observed only once compared between HTLV-1 and HTLV-2 (p<0.001, Mann-Whitney test).

In an earlier study, we showed that HTLV-1 infection is characterized by very large numbers of clones (over 4,000 unique integration sites [UIS] have been observed in 10 µg of PBMC-derived DNA) and that much of the load (in non-malignant cases) is composed of low abundance clones [23]. We confirmed this observation here for HTLV-1, but the clonal distribution in HTLV-2 infection showed several marked differences (Figure 3A). Significantly lower numbers of unique integration sites were identified in samples from HTLV-2-infected individuals (median 16 UIS) than those from HTLV-1-infected individuals (median 766 UIS; Figure 3B). Using the recently developed biodiversity estimator ‘*DivE*’ (Laydon et al., manuscript submitted), HTLV-1-infected subjects were estimated to carry a median of 31,710 distinct clones in the blood (consistent with previous estimates for HTLV-1), whereas HTLV-2-infected subjects were estimated to carry a median of only 976 clones (p<0.001, Mann-Whitney test; Figure 3C).

The distribution of proviral load across identified clones also differed significantly between HTLV-1 and HTLV-2. To compare the two viruses, we used the oligoclonality index [23], a parameter based on the Gini index, as a measure of dispersion describing the magnitude of unevenness of a frequency distribution. The oligoclonality index ranges between 0 and 1, where a value of 0 represents a distribution in which each clone constitutes an equal share of the proviral load, and 1 represents an upper bound where the load is effectively made up by a single clone. A median oligoclonality index of 0.34 was observed in non-malignant HTLV-1 carriers, consistent with our previous findings. In contrast, the oligoclonality index was remarkably variable between HTLV-2-infected individuals, and on average significantly higher than in HTLV-1-infected individuals (median 0.73, p<0.001, Mann-Whitney test; Figure 3D). Furthermore, the proportion of singletons (clones identified only once) was significantly lower in HTLV-2 infection (median = 1.96%) than in HTLV-1 infection (median = 37.17%, p<0.001, Mann-Whitney test; Figure 3E).

### Features of clonal expansion and proviral load in HTLV-2 infection

**Figure 4:**
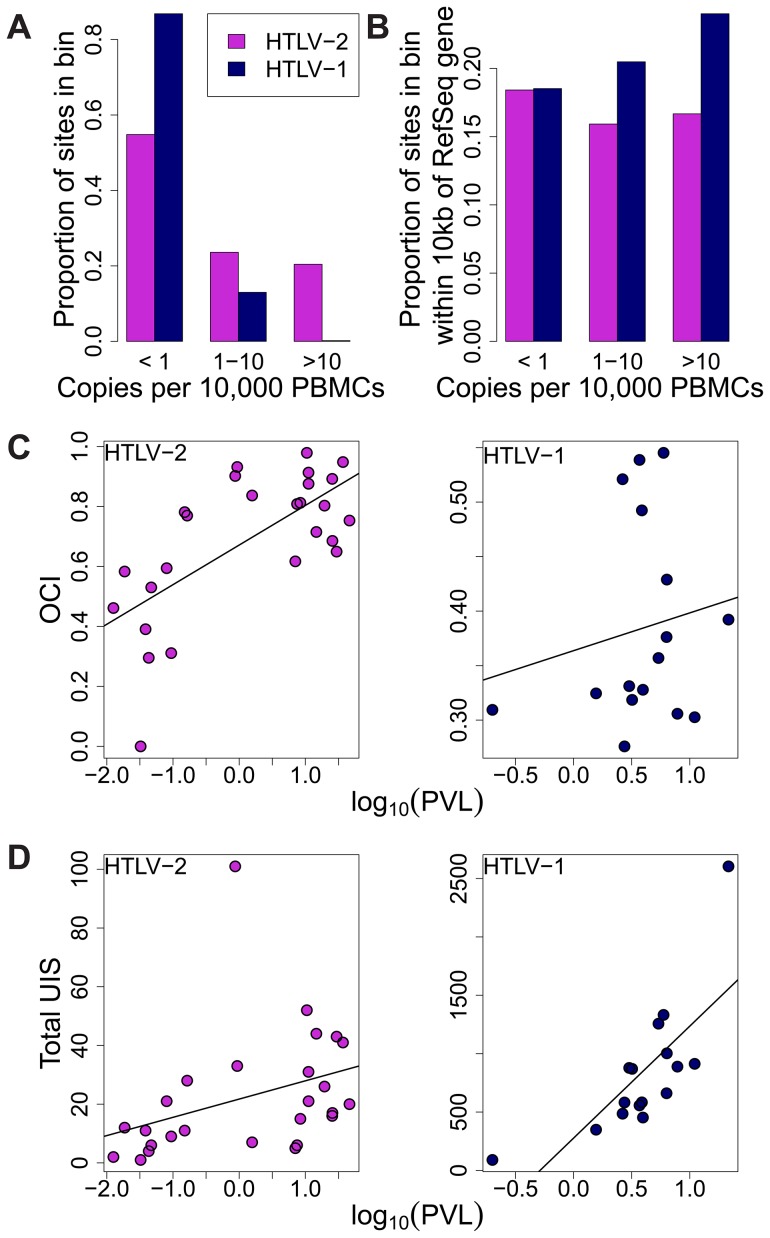
HTLV-2 integration site and clonal expansion. (A) The distribution of integration sites according to clonal abundance. Abundance was quantified by the number of copies estimated in a clone per 10,000 PBMCs (based on relative abundance and proviral load). Abundance bins are defined on a logarithmic scale. (B) The proportion of sites within 10 kb of a RefSeq gene for each abundance bin. A significant positive trend (p = 0.04, chi-squared test for trend) was detected for HTLV-1 but not for HTLV-2. (C) Oligoclonality index (OCI) versus log_10_(proviral load) for each virus. A strong positive correlation (p = 0.0015, Spearman's test) was detected between these parameters for HTLV-2 but not for HTLV-1(p = 0.681, Spearman's correlation). (D) The total number of unique integration sites (UIS) identified in each PBMC sample versus log_10_(proviral load) for each virus (p<0.001 for HTLV-1, p = 0.0019 for HTLV-2, Spearman's test).

The difference in clonal distribution between HTLV-1 and HTLV-2 was also apparent when measuring the absolute abundance of each clone as copies per 10,000 PBMCs (Figure 4A). In particular, highly expanded clones (i.e. those that each made up more than 0.1% of PBMCs) represented 20% of all HTLV-2 clones but only a fraction of all HTLV-1 clones (0.18%).

We reported previously that the genomic environment flanking the integration site appears to play a role in determining the equilibrium abundance of a given clone in vivo [23], [24]. The positive effect of integration within 10 kb of a RefSeq transcription start site on the abundance of HTLV-1 clones was observed here again (p = 0.04, chi-squared test for trend), but there was no correlation between the abundance of HTLV-2 clones and the proximity of a RefSeq gene (Figure 4B).

Whereas there was no significant correlation between the oligoclonality index and the proviral load in HTLV-1 infection (p = 0.681, rho = 0.112, Spearman's test), we observed a highly significant positive correlation (p = 0.0015, rho = 0.599, Spearman's test) between these parameters in HTLV-2 infection (Figure 4C). In contrast, the proviral load in HTLV-1 infection was more strongly correlated with the total number of clones (p<0.001, rho = 0.785, Spearman's test) compared to that in HTLV-2 (p = 0.0019, rho = 0.578, Spearman's test; Figure 4D).

### Clonal distribution in HTLV-2 infection does not change over time

**Figure 5:**
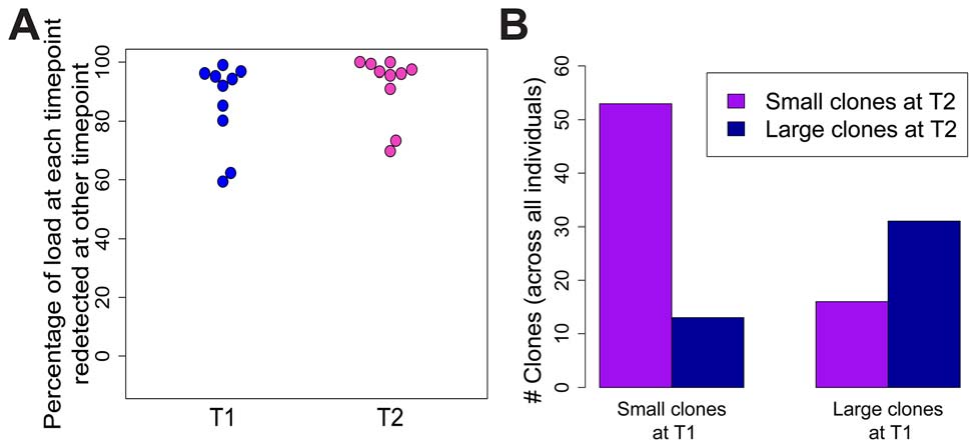
HTLV-2 expanded clones are long-lived and stable. Integration sites identified in PBMCs from an early time-point (T1, median = 9.9 years) were compared to those identified in the same HTLV-2-infected subjects at the present time-point (T2). (A) The percentage of proviral load (cumulative relative abundance) at each time-point represented by clones also present at the other time-point. (B) For clones found at both time-points, expanded clones (>1% of load) at any one time-point were significantly more likely to be expanded at both time-points (p<0.001, Fisher's exact test).

To test whether the expanded clones observed in HTLV-2 infection were long-lived, we analysed integration site content in samples collected at an earlier time point (range = 7.5 to 14.4 years, median = 9.9 years) from 10 of the HTLV-2-infected individuals in our cohort. The vast majority of the load (median = 96%) at the later time-point was represented by clones already present at the earlier time-point (Figure 5A). These clones did not change significantly in terms of their relative abundance; those representing >1% of the load at the later time-point were significantly more likely to make up >1% of the load at the earlier time-point and vice versa (p<0.001, OR = 7.73, Fisher's exact test; Figure 5B).

## Discussion

Both HTLV-1 and HTLV-2 infect the susceptible host by the same routes, and propagate within the host by the same two non-mutually-exclusive routes: the infectious route, which results in proviral integration at a new genomic site; and the mitotic route, where the provirus is replicated passively when the infected cell undergoes DNA replication and mitosis. It therefore benefits these viruses to drive proliferation of the infected cell. Indeed, the Tax proteins from both HTLV-1 and HTLV-2 have been shown in vitro to accelerate progression through the cell cycle, inhibit apoptosis and transform cells [3], [32], [33]. Consistent with these in vitro observations, we showed previously using metabolic labelling that cells spontaneously expressing the HTLV-1 Tax protein ex vivo proliferate faster in vivo [34].

HTLV-1 infection causes a chronic or acute T-lymphocytic malignancy in up to 5% of infected individuals [35], [36]; however, HTLV-2 is not unequivocally linked to a T-cell malignancy. Compared with the HTLV-1 Tax protein, HTLV-2 Tax is more dependent on IL-2 for the transformation of cells in culture [37]. This observation led to the suggestion that HTLV-2 would cause less in vivo proliferation of infected cells than HTLV-1, which in turn would decrease the oncogenic potential of the virus [26]. However, two lines of evidence go against this model. The first is the recent finding that HTLV-2 Tax has a greater in vitro immortalization capacity than HTLV-1 Tax in primary human T cells [38], [39]. The second is the finding reported here that HTLV-2 infection in vivo results in a small number of highly expanded T-cell clones (Figure 3). Although non-malignant HTLV-1 infection can result in the preferential expansion of certain clones, including clones that contain the provirus at genomic sites with particular characteristics [23], [24], HTLV-2 infection is capable of driving infected T-cells to proliferate selectively, generating clones which are often more highly expanded than those observed in non-malignant HTLV-1 infection (Figure 4A). The resulting clone frequency distribution in HTLV-2 infection is more similar to that observed in Adult T-cell Leukemia/Lymphoma (ATLL) patients than in non-malignant HTLV-1 infection (compare, for example, Figure 3D here with Figure 2B in [23]). That is, HTLV-1 infection is characterized by a large number of distinct clones in the circulation, while HTLV-2 infection is confined to a small number of highly expanded clones (Figure 3)

The host genomic environment flanking HTLV-2 integration sites in vivo closely resembles that of HTLV-1 integration sites. Similarities are evident at the nucleotide and the chromosome levels, and when examining particular genomic features known to be more frequent in proximity to HTLV-1 integration sites than expected by chance [24] (Figure 2). This observation is likely to result from the similarity between the HTLV-1 and HTLV-2 integrases, leading to shared targeting preferences during initial infection and integration. Since the samples analysed here (for both viruses) were drawn from patients infected for many years, this result suggests that there are also similar selection forces acting upon the infected cells in vivo in the two respective infections; the major selection force that differs between infected individuals is likely to be the acquired immune response, in particular cytotoxic CD8^+^ T-lymphocyte (CTL) activity [40].

The selection forces that act upon HTLV-1-infected clones have been the subject of many previous studies [41]. Two principal opposing forces govern the abundance of each clone in vivo: the ability of the clone to proliferate (e.g. through Tax-mediated cell proliferation or through antigen-mediated activation), and the susceptibility of the clone to elimination by CTL-mediated cell killing. We recently demonstrated that the genomic environment at the proviral integration site is associated with the clonal expansion in HTLV-1-infected individuals and with the tendency of a given clone to express the HTLV-1 Tax protein [23], [24]. Further, cells that spontaneously expressed the HTLV-1 Tax protein belonged more frequently to low-abundance clones in vivo compared with non-Tax-expressing cells, suggesting that the expression of this dominant T-cell immunogen [42], [43] limits proliferation in vivo. We suggest that this limited proliferation of Tax-expressing cells is due to counter-selection by the abundant, chronically activated Tax-specific CTLs.

The immune response to HTLV-2 proteins is less well understood. Oliveira and colleagues showed that high frequencies of CTLs specific for HTLV-2 Tax can be found in the circulation of HTLV-2 carriers [44]. Thus, while the genomic site preferences for HTLV-1 were mirrored in HTLV-2 infection, it is surprising that the integration site plays a lesser role as a determinant of clonal expansion in HTLV-2-infected individuals (Figure 4B).

Although abundant clones (absolute abundance >10 cells per 10,000 PBMCs) represent only a small fraction of all infected clones in HTLV-1 infection, they represented approximately 20% of all HTLV-2 clones in this study (Figure 4A). There are two possible explanations for this discrepancy: either HTLV-2 clones are not controlled as efficiently as HTLV-1 clones by the immune response, or there is an unidentified driver (in addition to the virus itself) that determines the proliferation of HTLV-2 clones. One potential additional driver is antigenic stimulation of the infected cells.

Regardless of the forces that drive this vigorous clonal proliferation of HTLV-2-infected cells, the observed correlation between the oligoclonality index and proviral load in HTLV-2 infection (a correlation not observed in non-malignant HTLV-1 infection, see Figure 4C) suggests that clonal proliferation plays a greater role in determining the viral burden of HTLV-2 than it does in HTLV-1. Conversely, in HTLV-1 infection, the total number of clones is more important as a determinant of viral burden (Figure 4D), consistent with previous observations [23], [28]. We conclude that the proviral load in HTLV-1 infection – and therefore the risk of both inflammatory and malignant disease – is determined primarily by the extent of infectious spread of the virus, and that oligoclonal proliferation per se, contrary to previous belief, does not contribute to HTLV-1-associated diseases. It remains an important question whether infectious spread is mainly confined to the early stages of infection or whether it persists indefinitely, with continual formation and destruction of many low-abundance clones. Work is now in progress to quantify the ratio of infectious spread to mitotic spread in these two infections.

Given the observations that HTLV-2-infected clones proliferate to a greater extent than many HTLV-1-infected clones in vivo, and that HTLV-2 shows transformation potential in vitro [3], [45], it is puzzling that HTLV-2 is not associated with a T-cell malignancy. One potential explanation is that the expansion of HTLV-2 clones in vivo is short-lived, and that major clones succeed each other over time. To test this possibility, integration sites and clonal distribution in PBMCs taken at an earlier time-point in the infection were compared to those identified ∼10 years later in 10 HTLV-2-infected individuals. Although there was no significant difference in the oligoclonality index or the total number of clones after correcting for the different numbers of proviruses detected (not shown), the bulk of the load at the later time-point (median = 96%, Figure 5A) belonged to clones already present at the earlier time-point, and that these clones principally maintained their expanded state over that period of time (Figure 5B). A similar observation was made in HTLV-1 infection by Gillet et al [23]. These observations reinforce the conclusion reached above that oligoclonal proliferation of infected T-cells in vivo does not in itself predispose to malignant disease in these retroviral infections.

To determine the proportion of the proviral load carried by CD4^+^ and CD8^+^ T-cells, we analysed the relative contribution of each identified clone to the load to calculate the cumulative contribution of each cell type. Using this method we found that HTLV-2 was primarily restricted to CD8^+^ T-cell clones (mean = 96.3%; Figure 1). The main limitation of this method is the sampling probability - i.e. the chance of redetection. If a clone is detected in unsorted PBMCs but not in the sorted sample it is not possible to attribute the clone to either CD4^+^ or CD8^+^ cells, or to distinguish the lack of redetection by chance from the possibility that the load is present in a different cell type (e.g. B cells [46]). However, since more abundant clones are more likely to be redetected in repeated experiments (N. Gillet and H. Niederer, unpublished observations), the fact that 70% of high-abundance clones (each constituting >1% of the load) were redetected compared with only 40% of low-abundance clones (each constituting <1% of load) in one of the cell-sorted populations suggests that low clone abundance, rather than an untested cell type, was responsible for the small fraction of the load not identified within either the CD4^+^ or CD8^+^ T-cell compartments.

It remains unclear what controls the proliferation of HTLV-2 clones in vivo, and what mechanisms underlie the difference in oncogenic potential of the two viruses. Possible factors include differences between HTLV-1 and HTLV-2 in the actions of the respective Tax protein [47] or the antisense proteins HBZ (HTLV-1) and APH-2 (HTLV-2) [48]. Also, CD4+ and CD8+ T cells may differ in their susceptibility to malignant transformation. A useful insight may be found by examining the clonal distribution of CD8^+^ cells infected with HTLV-1. A comparison between the clonal distribution of HTLV-1 and HTLV-2 in CD8^+^ cells may enable a distinction between effects due to infected cell phenotype and effects due to the differences in viral genome. This project is currently underway.

In summary, we report a comprehensive analysis of integration site preferences and clonal distribution in HTLV-2 infection. By comparison with similar data from HTLV-1-infected individuals, our results suggest an important distinction between virus-driven cell proliferation and virus-driven malignancy, and strengthen the conclusion that oligoclonal proliferation per se does not predispose to malignant transformation.

## Methods

### Ethics statement

UK blood samples were obtained through the Communicable Diseases Tissue Bank at Imperial College, approved by the UK National Research Ethics Service (NRES reference 09/H0606/106). Samples, with data linkage, were donated by HTLV-1 or HTLV-2-infected subjects attending the National Centre for Human Retrovirology, St Mary's Hospital, Imperial College Healthcare NHS Trust, London after giving written informed consent. HOST Study approved by the University of California San Francisco Committee on Human Research.

### Patients and cells

Cryopreserved PBMCs from 16 HTLV-1-infected and 28 HTLV-2-infected individuals were used in this study (Supplementary Table S1). Twenty-six HTLV-2-infected subjects were recruited to the HOST cohort, a long-term study of outcomes of HTLV-1 and HTLV-2 infection [49]. Two HTLV-2 and all 16 HTLV-1-infected subjects were Communicable Diseases Tissue Bank donors. Proviral load data on the HTLV-1-infected individuals were reported previously [50]. Genomic integration sites in 6 of the 16 HTLV-1-infected individuals were also studied previously, albeit at a distinct time-point in each case [23].

All DNA extractions were carried out using a DNeasy Blood & Tissue Kit (Qiagen) according to the manufacturer's protocol.

### Quantification of proviral load

HTLV-2 proviral load was quantified as reported elsewhere [51]. We used the proviral load for each patient at the nearest available time-point, because the proviral load was not always known for each at the same time-point at which clonality was analysed, and because HTLV-2 proviral load is reported to be stable over time [52]. Consistent with this assumption, our findings were not qualitatively altered by using a proviral load measurement from a different time-point.

### Cell sorting

Cells were thawed and washed, then surface-stained with directly-conjugated monoclonal antibodies specific for CD3, CD4 and CD8. Flow cytometric sorting was conducted to high purity (>98%) using a custom-modified FACSAria II (BD Biosciences). Lymphocytes were pre-gated on CD3, then sorted as CD4^+^CD8^−^ and CD4^−^CD8^+^ populations. Data were analysed with FACSDiva v6 software (BD Biosciences). For each sample, DNA was extracted from both sorted populations and from a separate aliquot of unsorted PBMCs.

### Integration site analysis

Ligation-mediated polymerase chain reaction (LM-PCR) primer binding site sequences were determined from Sanger-sequenced PCR amplicons (HTLV-1 primers: 5LTRfw –CTCGCATCTCTCCTTCACG, 5LTRrev – CTGGTGGAAATCGTAACTGGA; HTLV-2 primers: H2LTRfw – GACTCACCTTGGGGATCCAT, H2LTRrev – TTAGCCAAATGGGCGTTTTA). Identification of integration sites was performed via LM-PCR followed by high-throughput sequencing as described previously [23], using the HTLV-2-specific forward primers: H2B3 – AAGGGCTAGGGCTTCCTGAACCTC and H2B5 – CTATAGGCAGGCCCGCCCCAGGAG (or variants thereof according to defined LTR polymorphisms).

Prepared libraries were mixed and sequenced using either a Genome Analyzer II or a HiSeq System (Illumina). The resulting sequences were aligned to the human genome reference (hg18, excluding haplotypes, randoms and mitochondrial DNA) and HTLV-1/2 upstream sequence using the eland_pair implementation of CASAVA 1.8.2 (Illumina).

### Bioinformatics

Integration sites were quantified by enumerating unique shear sites as described previously [23]. Bins of absolute clonal abundance were determined by the number of copies per 10,000 PBMCs (i.e. relative clonal abundance multiplied by proviral load). In flow-sorted samples, clones were attributed exclusively either to the CD4^+^ or CD8^+^ cell population. Six clones were initially detected in both CD4^+^ and CD8^+^ cells; in these cases the clone was ascribed to the cell type with the greater number of proviruses of that clone.

In silico sites were derived by random selection of 190,000 sites from the human genome (hg18). To eliminate any potential bias due to alignment limitation, the DNA sequences at those sites were generated using Galaxy [53], [54] and back-aligned to the human genome using the same pipeline.

Annotations to the human genome (hg18) were retrieved as described previously (see Table S3 in [24]) and compared to integration sites using the R package hiAnnotator (http://malnirav.github.com/hiAnnotator), kindly provided by Nirav Malani and Frederic Bushman (University of Pennsylvania, Philadelphia, USA).

The *DivE* estimator was used to estimate the total number of clones, in addition to those observed. *DivE* involves fitting many mathematical models to individual-based rarefaction curves (Laydon et al., manuscript submitted). Here, individual-based rarefaction curves depict the expected number of clones against the number of infected cells sampled. Four numerical criteria are used to score each model in terms of how consistently it reproduces the total observed rarefaction curve from nested subsamples thereof. Using the geometric mean, estimates from the best-performing models are aggregated to produce the final estimate. Samples with near-linear rarefaction curve (i.e. where the curvature was less than 0.1(Laydon et al., manuscript submitted) were excluded from the analysis. Such curves imply a (biologically impossible) linear relationship between the number of infected cells and the number of clones, which is indicative of severe under-sampling. Prohibitively small sample sizes of less than 150 proviruses were also excluded from the analysis. *DivE* requires an estimate of the number of cells in the blood N_blood_, for which we assume: (i) a circulating blood volume of 5L; (ii) a PBMC count of 3×10^9^ L^−1^; and (iii) each infected T-cell carries a single copy of the provirus [55]. The number of PBMCs is thus assumed to be 5×3×10^9^. Proviral load (PVL) is defined as the number of viral copies per 100 PBMCs. Therefore, N_blood_ is given by PVL×5×3×10^9^.

### Statistics

Statistical analysis was carried out using R version 2.15.2 (http://www.R-project.org/). The Gini coefficient was calculated using the reldist R package ([56]; http://CRAN.R-project.org/package=reldist). Two-tailed non-parametric tests including the Mann-Whitney and Fisher's exact test were used for all comparisons.

## Supporting Information

Figure S1 **Experimental output per tissue type.** High-throughput sequencing analyses of integrated HTLV-2 proviruses are shown for each tissue type. CD4^+^/CD8^+^ cells – PBMCs from 28 HTLV-2-infected subjects were sorted by flow-cytometry into distinct CD3^+^CD4^+^CD8^−^ and CD3^+^CD4^−^CD8^+^ populations. PBMCs – unsorted PBMCs from 28 HTLV-2-infected individuals. PBMC early – unsorted PBMCs isolated previously from 10 of the HTLV-2-infected subjects. (A) The total number of sequencing reads for each HTLV-2 sample. (B) The total number of infected cells (distinct proviruses) identified in each HTLV-2 sample. (C) The total number of unique integration sites identified in each HTLV-2 sample.
(TIF)

Figure S2 **Integration bias at the sequence level.** Nucleotide sequences at the genomic sites of HTLV-2 integration are summarized by position and base. The arrow denotes the position of proviral integration. Base 1 is the first nucleotide following the integrated provirus. For each position, the relative proportion of sites containing each base is noted. Remarkably favoured (red) or disfavoured (blue) bases compared to random (in silico) sites are highlighted.
(TIF)

Table S1 **Details of subjects in study.**
(DOCX)
