# Supplementary figures and images for: Clonality of HTLV-2 in Natural Infection
Source: PLoS Pathog. 2014 Mar 13;10(3):e1004006. doi: 10.1371/journal.ppat.1004006 (PMC3953477; doi:10.1371/journal.ppat.1004006)

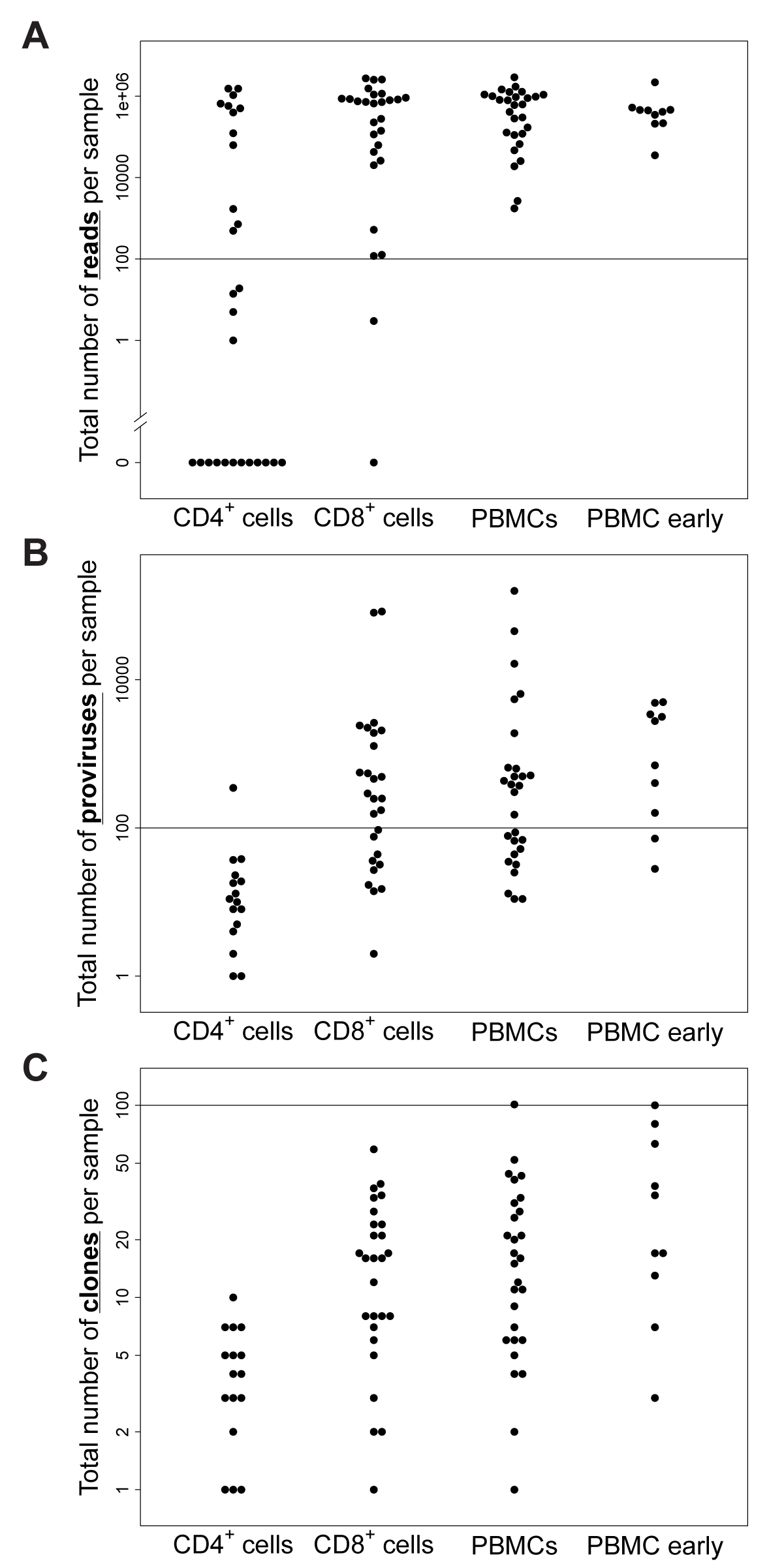

Supplement: Figure S1 — Experimental output per tissue type. High-throughput sequencing analyses of integrated HTLV-2 proviruses are shown for each tissue type. CD4+/CD8+ cells – PBMCs from 28 HTLV-2-infected subjects were sorted by flow-cytometry into distinct CD3+CD4+CD8− and CD3+CD4−CD8+ populations. PBMCs – unsorted PBMCs from 28 HTLV-2-infected individuals. PBMC early – unsorted PBMCs isolated previously from 10 of the HTLV-2-infected subjects. (A) The total number of sequencing reads for each HTLV-2 sample. (B) The total number of infected cells (distinct proviruses) identified in each HTLV-2 sample. (C) The total number of unique integration sites identified in each HTLV-2 sample. (TIF) [file ppat.1004006.s001.tif]

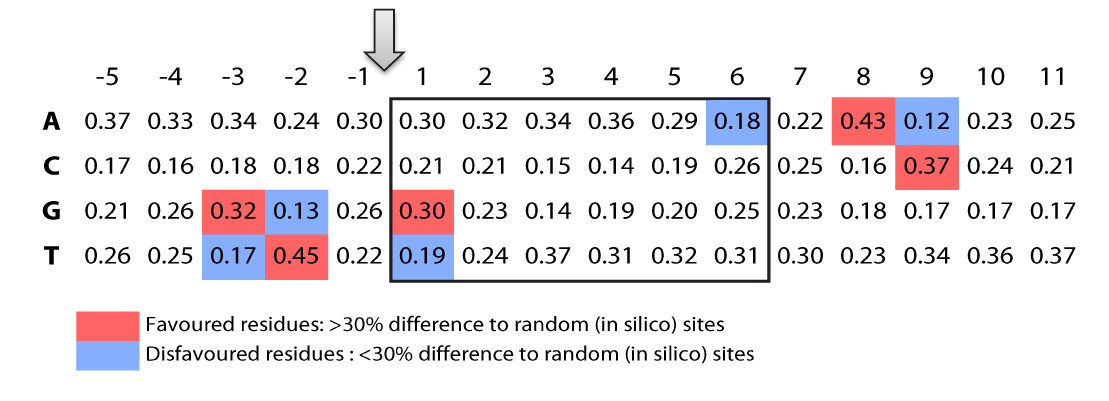

Supplement: Figure S2 — Integration bias at the sequence level. Nucleotide sequences at the genomic sites of HTLV-2 integration are summarized by position and base. The arrow denotes the position of proviral integration. Base 1 is the first nucleotide following the integrated provirus. For each position, the relative proportion of sites containing each base is noted. Remarkably favoured (red) or disfavoured (blue) bases compared to random (in silico) sites are highlighted. (TIF) [file ppat.1004006.s002.tif]
