# Supplementary material for: Clonality of HTLV-2 in Natural Infection
Source: PLoS Pathog. 2014 Mar 13;10(3):e1004006. doi: 10.1371/journal.ppat.1004006 (PMC3953477; doi:10.1371/journal.ppat.1004006)
Supplement: Table S1 — Details of subjects in study. (DOCX) [file ppat.1004006.s003.docx]

**Table S1 – Details of subjects in study**

| Virus | Patient Code | Gender | Age | Total Reads | Total UIS | Total Proviruses | Proviral load | Known coinfections | Clinical outcome (HTLV-1) | Cohort | Reported elsewhere |
| --- | --- | --- | --- | --- | --- | --- | --- | --- | --- | --- | --- |
| HTLV-2 | 973 | F | 66 | 795571 | 2 | 52 | 0.01 |  | -- | HOST | NA |
| HTLV-2 | 237 | F | 53 | 119853 | 1 | 11 | 0.03 |  | -- | HOST | NA |
| HTLV-2 | 180 | F | 54 | 283856 | 21 | 311 | 46.42 |  | -- | HOST | NA |
| HTLV-2 | 565 | F | 68 | 1270096 | 6 | 44 | 0.05 |  | -- | HOST | NA |
| HTLV-2 | 1373 | M | 60 | 954193 | 11 | 67 | 0.04 | hcv | -- | HOST | NA |
| HTLV-2 | 98 | F | 60 | 975056 | 14 | 86 | 0.02 | hcv, syph | -- | HOST | NA |
| HTLV-2 | 121 | F | 46 | 606509 | 43 | 432 | 29.66 |  | -- | HOST | NA |
| HTLV-2 | 1171 | M | 46 | 1003319 | 33 | 5452 | 0.93 |  | -- | HOST | NA |
| HTLV-2 | 367 | M | 49 | 900079 | 17 | 45225 | 25.46 |  | -- | HOST | NA |
| HTLV-2 | 86 | M | 63 | 1288537 | 23 | 115 | 0.08 | hcv | -- | HOST | NA |
| HTLV-2 | 207 | F | 64 | 25602 | 7 | 16 | NA |  | -- | HOST | NA |
| HTLV-2 | 825 | M | 52 | 1465484 | 6 | 497 | 7.49 | hcv | -- | HOST | NA |
| HTLV-2 | 1264 | F | 53 | 171004 | 11 | 29 | 0.09 |  | -- | HOST | NA |
| HTLV-2 | 2M | F | 47 | 66818 | 21 | 650 | 11.18 |  | -- | NCHR | NA |
| HTLV-2 | 1047 | M | 48 | 1766 | 9 | 42 | 7.05 |  | -- | HOST | NA |
| HTLV-2 | 1359 | F | 54 | 46945 | 27 | 512 | 19.48 | hcv, hbv, syph | -- | HOST | NA |
| HTLV-2 | 1486 | M | 78 | 1720684 | 10 | 497 | 1.57 |  | -- | HOST | NA |
| HTLV-2 | 354 | F | 57 | 2919351 | 53 | 158171 | 10.53 |  | -- | HOST | NA |
| HTLV-2 | 599 | M | 65 | 2678 | 20 | 73 | 25.65 |  | -- | HOST | NA |
| HTLV-2 | 797 | M | 70 | 1092176 | 8 | 44 | NA |  | -- | HOST | NA |
| HTLV-2 | 1345 | M | 52 | 128203 | 6 | 15 | 0.04 | hcv, syph | -- | HOST | NA |
| HTLV-2 | 1038 | M | 45 | 1101421 | 31 | 1897 | 11.16 |  | -- | HOST | NA |
| HTLV-2 | 1135 | F | 48 | 300905 | 16 | 156 | 0.15 |  | -- | HOST | NA |
| HTLV-2 | 2P | F | 43 | 19018 | 17 | 380 | 8.41 |  | -- | NCHR | NA |
| HTLV-2 | 996 | M | 53 | 629314 | 42 | 16437 | 37.01 |  | -- | HOST | NA |
| HTLV-2 | 425 | F | 28 | 111525 | 45 | 389 | 14.65 |  | -- | HOST | NA |
| HTLV-2 | 316 | F | 52 | 412669 | 105 | 6438 | 0.87 |  | -- | HOST | NA |
| HTLV-2 | 112 | F | 49 | 814483 | 28 | 631 | 0.16 |  | -- | HOST | NA |
| HTLV-1 | TCJ | F | 43 | 4880 | 889 | 1448 | 7.83 |  | HAM/TSP | NCHR | NA |
| HTLV-1 | TCP | M | 71 | 4656 | 913 | 1428 | 11.01 |  | HAM/TSP | NCHR | NA |
| HTLV-1 | HAP | M | 72 | 43078 | 878 | 1499 | 3.01 |  | AC | NCHR | NA |
| HTLV-1 | HCH | F | 67 | 35110 | 1003 | 2027 | 6.34 |  | AC | NCHR | NA |
| HTLV-1 | HDS | F | 59 | 50318 | 870 | 1480 | 3.19 |  | AC | NCHR | Gillet2011 |
| HTLV-1 | HBZ | F | 65 | 45316 | 453 | 757 | 3.95 |  | AC | NCHR | NA |
| HTLV-1 | HAY | F | 65 | 57594 | 1332 | 3563 | 5.95 |  | AC | NCHR | Gillet2011 |
| HTLV-1 | HBX | F | 53 | 33598 | 583 | 1496 | 3.87 |  | AC | NCHR | Gillet2011 |
| HTLV-1 | HFG | F | 63 | 71504 | 486 | 1529 | 2.64 |  | AC | NCHR | Gillet2011 |
| HTLV-1 | TAZ | M | 68 | 36489 | 2604 | 4874 | 21.52 |  | HAM/TSP | NCHR | Gillet2011 |
| HTLV-1 | TCI | F | 65 | 70730 | 349 | 569 | 1.56 |  | HAM/TSP | NCHR | NA |
| HTLV-1 | HAO | F | 48 | 61687 | 90 | 142 | 0.20 |  | AC | NCHR | NA |
| HTLV-1 | HFB | F | 43 | 54123 | 557 | 1854 | 3.69 |  | AC | NCHR | NA |
| HTLV-1 | TBR | F | 56 | 66595 | 1257 | 2280 | 5.38 |  | HAM/TSP | NCHR | Gillet2011 |
| HTLV-1 | TCF | F | 54 | 39841 | 582 | 873 | 2.74 |  | HAM/TSP | NCHR | NA |
| HTLV-1 | TCL | F | 54 | 34709 | 661 | 1266 | 6.31 |  | HAM/TSP | NCHR | NA |

**AC** - Asymptomatic Carrier

**HAM/TSP** - HTLV-1 associated myelopathy/tropical spastic paraparesis

**UIS** - unique integration sites

**Total proviruses** - total integrations (calibrated shear sites) across all UIS

**Total reads** - total sequencing reads across all UIS

**Proviral load** - proviral copies per 100 PBMCs

**hcv** - Hepatitis C Virus

**hbv** - Hepatitis B Virus

**syph** - Syphilis

**HOST** - HTLV outcomes study

**NCHR** - National Centre for Human Retrovirology at St Mary’s hospital, London UK

**Reported elsewhere** - clonality of this HTLV-1 infected subject (a different time-point) was reported previously (Gillet et al, 2011)
